# Comparative outcomes of gram-positive and gram-negative intraoperative infections in degenerative scoliosis: a 10-year propensity score-matched analysis

**DOI:** 10.1007/s43390-025-01234-5

**Published:** 2025-11-25

**Authors:** Yousaf B. Ilyas, Tania M. Aguilar, Hadeel M. Mansour, Kristina P. Kurker, Kaho Adachi, Morteza Sadeh, Nauman S. Chaudhry, Ankit I. Mehta

**Affiliations:** 1 University of Illinois College of Medicine at Chicago, 1853 W. Polk St., Chicago, IL 60612 USA; 2 Department of Neurosurgery, University of Illinois, 840 S Wood St., Chicago, IL 60612 USA; 3 Orlando Health Neuroscience Institute, 76 W Underwood St., Orlando, FL 32806 USA

**Keywords:** Scoliosis, Infection, Gram-positive, Gram-negative, Postoperative outcomes

## Abstract

**Purpose:**

In this study, we examine the characteristics of Gram-negative and Gram-positive infections following scoliosis corrective surgery, as well as the potential risk factors contributing to infection.

**Methods:**

We queried the Scoliosis Research Society (SRS) database from 2013 to 2023 to identify patients with degenerative scoliosis who developed postoperative infections. To account for comorbidities and minimize bias, 1:1 propensity score matching with replacement was performed. The total cohort sample was 706 (353 in each group), with 64% being female and a mean age of 67.9 years. The outcomes analyzed included infection duration, presence of neurological deficits, and mortality.

**Results:**

Gram-negative infections had higher rates of return to the operating room (95.2% vs 74.5%, *p* < 0.001), and re-insertion of instruments (10.5% vs 4.5%, *p* = 0.004). However, Gram-positive bacteria were associated with higher rates of antibiotic-related complications (4.3% vs 1.1%, *p* = 0.02).

**Conclusion:**

Gram-positive infections were associated with prolonged infection courses and higher mortality, while Gram-negative infections more often resulted in a return to surgery and neurological deficits. These findings suggest that Gram staining may provide an early and clinically meaningful distinction in risk stratification for patients with postoperative wound infections following scoliosis surgery.

## Introduction

Postoperative surgical site infections (SSIs) remain one of the most common and challenging complications following spinal surgeries, ranking third after pneumonia and urinary tract infections [1, 2]. SSIs can be classified into superficial, deep, and organ-space infections, depending on the depth and location of the infection [3]. Superficial SSIs involve the skin and subcutaneous tissues, deep SSIs extend to the fascia and muscle layers, while organ-space SSIs affect the surgical site beyond these layers. The reported incidence of SSI after spinal surgery ranges between 0.7 and 16.1% [4]. Among the common pathogens, *Staphylococcus aureus* and *Staphylococcus epidermidis* are the leading Gram-positive organisms responsible for SSIs, mainly due to their ability to form biofilms on spinal implants [5]. Although less frequent, Gram-negative bacteria such as* Pseudomonas aeruginosa*,* Escherichia coli*, and *Klebsiella pneumoniae* are associated with more severe infections, higher antibiotic resistance, and worse clinical outcomes [6, 7].

SSIs are particularly concerning in spinal deformity surgeries, such as scoliosis and kyphosis corrections, where multi-level instrumentation, longer operative times, and increased blood loss are common, contributing to an elevated risk of infection [8–10]. Patients with diabetes, obesity, or a history of previous SSIs are at an even greater risk of postoperative infection [11–13]. Beyond the immediate postoperative complications, SSIs are associated with prolonged hospital stays, delayed recovery, increased healthcare costs, and in severe cases, permanent neurological deficits or even mortality [8, 14, 15].

Despite the recognized significance of pathogen-specific infections, limited studies have systematically compared Gram-positive and Gram-negative SSIs specifically in the context of scoliosis surgery. A majority of the existing literature tends to group infections broadly without differentiating between the clinical implications of various organisms, focusing mainly on infection rates and general management strategies. Therefore it difficult to precisely characterize infection-specific risks, prognoses, and management challenges in the high-risk scoliosis population.

The management of spinal SSIs generally involves surgical debridement and intravenous antibiotic therapy during the acute phase; however, chronic infections may require implant removal and revision surgery [16]. Understanding the differences between Gram-positive and Gram-negative infections could help tailor preventive measures and therapeutic interventions more effectively. This study aims to address this gap in the literature by evaluating and comparing the impact of Gram-positive versus Gram-negative SSIs on postoperative outcomes in scoliosis surgery using a decade’s worth of data (2013–2023) from the SRS. Through this analysis, we aim to offer a more detailed understanding of infection-specific outcomes, guiding more informed prevention and management strategies in spinal deformity surgery.

## Methods

The Scoliosis Research Society Database (SRSDB) was queried from 2013 to 2023 for patients who underwent surgery for degenerative scoliosis. Patients were then selected if they had an instance of a postoperative surgical site infection. The groups were then split into two groups based on infection category: Gram-positive (Staph Aureus MRSA, Staph Aureus MSSA, Staph Epidermidis) and Gram-negative (*E. coli*,* Pseudomonas*, and Proteus). Propensity score matching without replacement was implemented to reduce biases between the groups. Matching was done on age and different comorbidities, including smoking status, heart disease, kidney disease, neurological disease, pulmonary disease, obesity, and osteopenia (see Table 1). After propensity score matching on age and co-morbidities, there were 353 patients in each group, and Chi-squared tests and student T-tests were run to compare postoperative outcomes amongst the groups. Finally, bacteria from both groups were further analyzed using pairwise comparisons to see if there were any differences among the individual bacteria in terms of postoperative outcomes. Bonferroni corrections were implemented to reduce the likelihood of false positives.

**Table 1:** Comorbidities post-matching

Comorbidity	Gram-negative (%)	Gram-positive (%)	*p* value	SMD
Alcohol abuse	0.0	0.9	0.247	0.1
Anemia	1.4	2.3	0.576	0.1
Cancer	0.3	2.8	**0.015**	0.2
Collagen vascular disease	0.3	1.4	0.219	0.1
Diabetes mellitus	7.7	13.3	**0.020**	0.2
Hypertension	10.8	21.8	** < 0.001**	0.3
Heart disease	7.1	7.4	1.000	0.0
Kidney disease	1.1	1.4	1.000	0.0
Liver disease	0.3	0.3	1.000	0.0
Neurologic disease	0.3	0.3	1.000	0.0
Obesity	9.9	12.5	0.340	0.1
Osteopenia	8.2	8.8	0.893	0.0
Pulmonary disease	5.4	4.5	0.729	0.0
Smoking	3.4	4.0	0.842	0.0

*p *-values less than 0.05 are bold

## Results

**Fig. 1:**
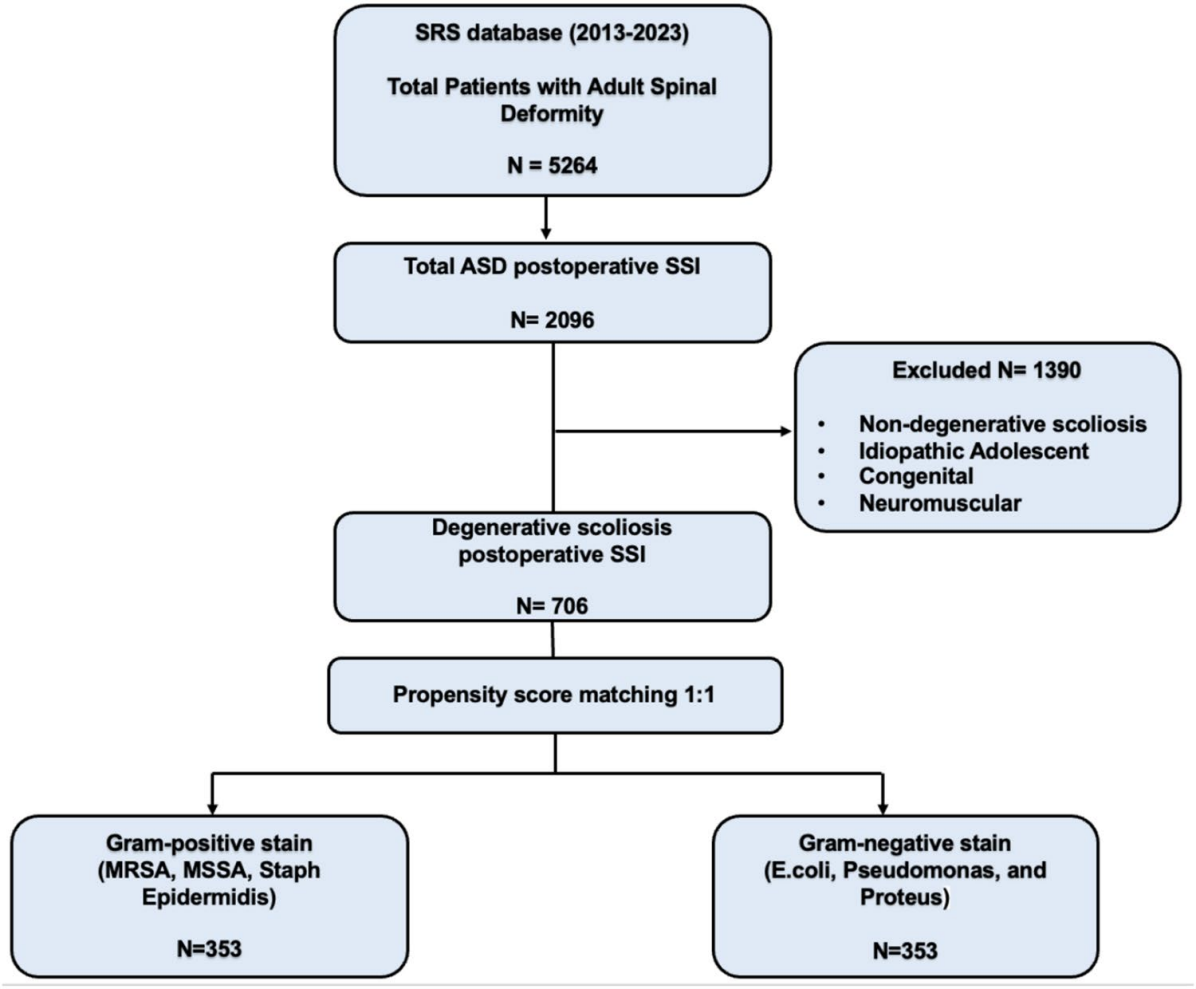
Inclusion Criteria

After matching, our sample size consisted of 706 patients with postoperative surgical site infections (SSIs) following degenerative scoliosis surgery were included after applying strict inclusion and exclusion criteria (Fig. 1). Each Gram-staining group had a sample size of 353, with balanced baseline demographics and comorbidities (Table 1). The mean ages for the Gram-positive and negative groups were 67 and 68 years respectively, and the most common comorbidities included hypertension (21.8% vs 10.8%), obesity (12.5% vs 9.9%), and diabetes (13.3% vs 7.7%). Aside from hypertension, balance was achieved for all comorbidities (Table 1).

When postoperative outcomes were compared, Gram-negative infections had higher rates of return to the operating room (95.2% vs 74.5%, p < 0.001), and re-insertion of instruments (10.5% vs 4.5%, p = 0.004). However, Gram-positive bacteria were associated with higher rates of antibiotic-related complications (4.3% vs 1.1%, p = 0.02). There was no significant difference between length of stay (21.4 vs 20.2 days, p = 0.29), or any of the other measured outcomes (Table 2).

**Table 2:** Outcomes of Gram-positive vs Gram-negative infections

Variable	Gram negative (%)	Gram positive %	*p *value
New post-operative neurologic deficit (Y/N)	7.4	6.0	0.546
Complications led to death (Y/N)	1.1	3.4	0.077
Sepsis (complication)	1.1	2.6	0.263
Respiratory complication (death section)	0.6	0.3	1.000
Multi-organ failure (death section)	0.6	0.9	1.000
Returned to surgery (Y/N)	95.2	74.5	**< 0.001**
Instrumentation removed (Y/N)	2.3	3.4	0.496
Loss of correction (Y/N)	2.0	3.7	0.257
Change in neurologic status (Y/N)	0.6	1.7	0.286
Complications from antibiotics (Y/N)	1.1	4.3	**0.020**
Instruments re-inserted (Y/N)	10.5	4.5	**0.004**

*p *-values less than 0.05 are bold

Organism-specific analysis revealed further differences (Table 3 and Table 4). Among Gram-positive organisms, MRSA was associated with higher rates of changes in neurologic status (5.9% vs 0.9% vs 0%, p = 0.04). However, there were no other significant differences amongst the Gram–positive organisms. There was considerably more variance in the Gram-negative group, with Proteus and unspecified bacteria having greater rates of return to surgery (100% and 93.9%, p = 0.0032) and length of stay (28.4 and 24.0 days, p = 0.0477) respectively. No significant differences were observed among Gram-negative organisms for loss of correction, neurologic complications, or antibiotic-related events.

**Table 3:** Intra-gram analysis for Gram-positive organisms

Variable	MRSA (%/mean ( ±) SD)	Staph aureus (%/mean ( ±) SD)	Staph epidermidis (%/mean ( ±) SD)	*p* value
New post-operative neurologic deficit (Y/N)	7.1%	2.6%	4.9%	0.3342
Complications led to death (Y/N)	8.2%	1.8%	4.9%	0.096
Sepsis (complication)	7.1%	0.9%	2.4%	0.0524
Respiratory complication (death section)	0.0%	0.9%	0.0%	0.5741
Multi-organ failure (death section)	1.2%	0.9%	0.0%	0.7912
Returned to surgery (Y/N)	81.2%	74.6%	65.9%	0.1663
Instrumentation removed (Y/N)	5.9%	1.8%	2.4%	0.2595
Loss of correction (Y/N)	4.7%	4.4%	2.4%	0.8261
Change in neurologic status (Y/N)	5.9%	0.9%	0.0%	**0.0434**
Complications from antibiotics (Y/N)	7.1%	1.8%	7.3%	0.1368
Instruments re-inserted (Y/N)	7.1%	4.4%	0.0%	0.2049
Length of stay	23.8 ( ±) 18.6	19.6 ( ±) 16.3	18.3 ( ±) 15.1	0.1595

*p *-values less than 0.05 are bold

**Table 4:** Intra-gram analysis for Gram-negative organisms

Variable	*E. coli* (%/mean ( ±) SD)	Other (%/mean ( ±) SD)	Proteus (%/mean ( ±) SD)	Pseudomonas (%/mean ( ±) SD)	*p* value
New post-operative neurologic deficit (Y/N)	3.9%	15.2%	8.3%	8.7%	0.3363
Complications led to death (Y/N)	7.7%	3.0%	0.0%	0.0%	0.3551
Complication: pulmonary embolism (death section)	1.9%	0.0%	0.0%	0.0%	0.7247
Sepsis (complication)	5.8%	3.0%	0.0%	0.0%	0.5371
Respiratory complication (death section)	3.9%	0.0%	0.0%	0.0%	0.4471
Multi-organ failure (death section)	1.9%	0.0%	0.0%	0.0%	0.7247
Returned to surgery (Y/N)	90.4%	93.9%	100.0%	65.2%	**0.0032**
Instrumentation removed (Y/N)	9.6%	12.1%	0.0%	4.4%	0.5123
Loss of correction (Y/N)	1.9%	6.1%	8.3%	4.4%	0.6879
Change in neurologic status (Y/N)	5.8%	6.1%	0.0%	4.4%	0.8506
Complications from antibiotics (Y/N)	7.7%	9.1%	0.0%	8.7%	0.7666
Instruments re-inserted (Y/N)	7.7%	15.2%	0.0%	0.0%	0.1313
Length of stay	18.9 ( ±) 10.6	24.0 ( ±) 17.2	16.7 ( ±) 9.3	28.4 ( ±) 19.9	**0.0477**

*p *-values less than 0.05 are bold

## Discussion

In this retrospective cohort study, we utilized data from the SRSDB between 2013 and 2023 to identify patients who underwent surgery for degenerative scoliosis and subsequently developed a surgical site infection. The patients with Gram-positive infections were propensity score matched with those with Gram-negative groups according to age and comorbidities, including smoking status, heart disease, kidney disease, neurological disease, pulmonary disease, obesity, and osteopenia, resulting in a balanced number of patients in each group. We then compared postoperative outcomes between the two groups and performed pairwise comparisons between individual bacterial species. Our findings show distinct differences based on the type of infection. Gram-positive infections were associated with significantly greater rates of loss of correction, longer infection duration, and higher mortality, whereas Gram-negative infections were more strongly associated with return to the operating room and new neurological deficits. We also found that MRSA had more antibiotic-related complications compared to MSSA, and both MRSA and MSSA had higher rates of return to surgery compared to* Staphylococcus epidermidis*.

Our findings highlight the importance of considering the infecting organism when managing surgical site infections in patients undergoing deformity correction surgery for degenerative scoliosis. Gram-positive infections, particularly those caused by* Staphylococcus aureus* (*S. aureus*), were associated with significantly higher rates of loss of correction, prolonged infection duration, and increased mortality. Importantly, “loss of correction” was recorded in the SRS database only as a categorical variable (present/absent), without radiographic measures such as Cobb angle or deformity plane, which limits interpretation of this complication. Infections following spinal surgery have been linked to the development of pseudoarthrosis, a major complication frequently requiring surgical revision [17]. Hollern et al. reported a substantial association between surgical site infections and pseudoarthrosis, with incidence rates ranging from 30 to 85% [18]. Moreover, their study highlighted that the predominant pathogens isolated in these pseudoarthrosis cases were Gram-positive bacteria, specifically staphylococcal species, including MRSA, reinforcing our findings that Gram-positive infections significantly contribute to structural complications. Similarly, another study identified S. aureus as a leading cause of SSIs in spine procedures, responsible for nearly half (49.3%) of all reported infections and a pooled infection rate of 1% [19]. These Gram-positive infections have also been associated with increased mortality in spinal surgery patients, with reported rates ranging from 1.1 to 2.3%, and often requiring aggressive treatment such as surgical debridement and extended antibiotic usage [19, 20]. Standard prophylaxis for spine surgery typically includes cefazolin, with vancomycin used for MRSA carriers or patients with beta-lactam allergies, and gentamicin added for Gram-negative coverage in high-risk patients; antibiotics given within an hour before incision, with intraoperative redosing as needed [21]. However, one study found that 54% of surgical site infection cases involved either exclusively Gram-negative organisms or a combination of Gram-negative and Gram-positive bacteria in multilevel spinal fusion surgeries, raising the question of whether standard prophylactic antibiotic regimens offer sufficient coverage in these complex procedures [20]. Karamian et al. further demonstrated that infections caused by Gram-negative or mixed organisms were associated with a greater treatment burden, including long duration of IV antibiotic treatment and multiple surgical debridements [22]. These findings reinforce the importance of considering the infecting organism when managing SSIs, as different bacterial types carry distinct clinical burdens and may require aggressive management following complex spine surgeries. Our study also underscores the fact that Gram-positive infections were strongly associated with higher antibiotic-related complications, suggesting that different prophylactic strategies may be warranted in high-risk cases.

In our analysis of Gram-positive organisms, important differences emerged between S. aureus strains. Although MRSA and MSSA demonstrated similar impacts on most postoperative outcomes, MRSA was associated with a significantly higher rate of change of neurologic deficit. Furthermore, although the majority of outcomes didn’t reach statistical significance, MRSA had the highest rate of nearly every adverse postoperative outcome measured.. These findings underscore the added clinical complexity that MRSA introduces, not only due to its antimicrobial resistance but also because of the increased risk of treatment-related toxicity and more intensive surgical management. Patel et al. previously described how surgical site infections involving S. aureus were associated with greater healthcare utilization, including longer hospital stays and more frequent reoperations [19]. Our findings build on this by highlighting that MRSA may be the main driver of these burdens among S. aureus infections. Given the challenges MRSA poses, preoperative strategies focused on early identification and prevention are essential. Recent evidence has shown that implementing MRSA-targeted prophylaxis in spine surgery, such as vancomycin or intranasal mupirocin, can reduce postoperative infection rates [23]. Additionally, guidelines from the North American Spine Society emphasize the value of routine preoperative MRSA screening to identify high-risk patients and guide decolonization protocols [24]. Taken together, these findings highlight the need for organism-specific planning and surveillance to reduce complications and improve outcomes in degenerative scoliosis patients undergoing spine surgery. Although we performed organism-level analyses, the absence of antibiotic resistance profiles and biofilm formation data in the SRSDB limited our ability to evaluate mechanistic contributors to these outcomes.

The findings of this study highlight several avenues for future research and clinical application in the care of patients undergoing surgery for degenerative scoliosis. First, prospective studies are warranted to validate the associations between bacterial etiology and specific postoperative complications within this patient population. Since Gram-negative infections and MRSA were tied to more severe outcomes in our analysis, future work should look at whether broader or more tailored antibiotic strategies could help minimize complications associated with postoperative infections. It would also be valuable to explore the long-term effects of infection-related loss of correction, particularly in cases caused by S. aureus, where neurologic outcomes may be more significantly impacted. Future studies should also incorporate standardized CDC-based definitions of SSI, infection severity grading, intraoperative factors such as surgical complexity and blood loss, and radiographic measures of correction loss. The lack of significant differences amongst Gram-positive pathogens highlights the potential benefit for implementation of a standardized prophylactic protocol for spine surgery patients. Finally, the development of clinical prediction models that take into account patient comorbidities, infection type, and surgical complexity could help identify high-risk patients early and guide more personalized postoperative care.

## Limitations

This study has several important limitations. As a retrospective study, it carries inherent biases, including the inability to fully randomize patients, albeit propensity score matching was used to reduce confounding variables. However, unmeasured factors such as surgical technique, implant type, intraoperative contamination, and perioperative antibiotic regimens were not captured in the SRSDB. The absence of these variables limits our ability to fully account for all sources of variation in postoperative recovery. Additionally, the definition of surgical site infection was determined by the treating surgeon rather than using standardized CDC criteria, and infections were generally recorded within 90 days postoperatively; this heterogeneity may have influenced case inclusion.

Additionally, key infection characteristics, such as severity, timing of onset (early vs. late SSI), and microbiological resistance profiles were not available, limiting the clinical depth of the analysis. Information regarding biofilm formation, which plays a key role in spinal implant infections, was also unavailable. Treatment specific details, including choice of antibiotic treatment, duration of therapy, and surgical debridement protocols, were also not recorded. Moreover, while we assessed patients with diagnosed SSIs, undiagnosed or subclinical infections were not included, potentially underestimating the true burden of these infections.

Furthermore, the most common comorbidity was neurologic disease, but further detail was not available to account for the severity of disease which may have impacted results. While the dataset was cleaned to focus exclusively on adult degenerative scoliosis cases, residual heterogeneity may still exist, and the original analysis suggested possible inclusion of younger patients, which has now been addressed in the revision. While loss of correction was analyzed, details related to that loss, including degree of loss, were unavailable for further analysis, as it was only coded as a binary outcome rather than a radiographic measurement.

Finally, the SRSDB did not include complete demographic information for all patients, which limited our ability to analyze how factors such as race, ethnicity, or socioeconomic status may influence infection risk and outcomes, warranting further investigation. In addition, while organism-level analyses were performed in this revision, antibiotic resistance patterns could not be evaluated due to absence of sensitivity data, and this should be a focus for future prospective studies.

## Conclusion

In conclusion, this study shows that the type of bacterial pathogen has a great influence on postoperative outcomes following scoliosis surgery. Gram-positive infections, while being most common, were associated with higher rates of antibiotic-related complications, suggesting greater resistance to medication. In contrast, Gram-negative infections, although less frequent, were associated with higher rates of revision surgery and need for re-insertion of instrumentation, suggesting a more aggressive disease course. At the organism level, MRSA showed higher rates of change in neurological status, and also had the highest proportion of negative postoperative outcomes. Proteus was demonstrated to be the most virulent organism by having the longest length of stay and reoperation rates among Gram-negative organisms. However, unspecified bacteria was the second most virulent in the Gram-negative group, suggesting further research needs to be done across different Gram-negative species to determine virulence. These findings emphasize the need for surgeons to consider the individual infecting organism when evaluating prognosis and select the appropriate management strategies for postoperative spinal surgery care.

## Data Availability

We utilized data from the Scoliosis Research Society (SRS) Database, which is available for SRS members.
